# Effect of exercise based prehabilitation on postoperative pain in breast cancer patients: a systematic review with meta-analysis

**DOI:** 10.1007/s12282-026-01899-4

**Published:** 2026-07-30

**Authors:** Asier del Arco, Celia García-Chico, Kayvan Khoramipour, Arkaitz Castañeda-Babarro, Alejandro Santos-Lozano, Jose Pinto-Fraga

**Affiliations:** 1 Health, Physical Activity and Sports Science Laboratory, Department of Physical Activity and Sports, University of Deusto, Bilbao, Spain; 2 Health Sciences, i+HeALTH Strategic Research Group, Miguel De Cervantes European University, Valladolid, Spain

**Keywords:** Preoperative intervention, Physical therapy, Neoplasm, Surgery

## Abstract

**Background:**

Postoperative pain is a common complication following breast cancer surgery, adversely influencing recovery and quality of life. Exercise-based prehabilitation, initiated before surgery to enhance physical readiness, has shown benefits in other surgical settings. However, its specific impact on postoperative pain in breast cancer patients remains unclear.

**Methods:**

This systematic review and meta-analysis evaluated the effectiveness of exercise-based prehabilitation in reducing postoperative pain in women undergoing breast cancer surgery. Following PRISMA guidelines, a comprehensive search was conducted in PubMed, Scopus, and Web of Science up to June 2025 (search conducted prior to submission). Eligible studies were randomized controlled trials (RCTs) comparing exercise-based prehabilitation with usual care or educational interventions. The primary outcome was pain at one month post-surgery. Risk of bias was assessed using the Cochrane RoB 2 tool, and methodological quality via the TESTEX scale.

**Results:**

Six RCTs (*n* = 519) were included in the qualitative synthesis, and three (*n* = 157) in the meta-analysis. Interventions varied in duration (1–9 weeks), modality (aerobic, resistance, mobility, stretching), and supervision. Pain was measured using tools such as VAS, NPRS, EORTC QLQ-C30 and SPADI. Meta-analysis showed a small, non-significant effect (SMD = -0.24; 95% CI: -0.55 to 0.08; *p* = 0.14). Heterogeneity was statistically low (I² = 0%; 95% CI: 0% to 79%), although this estimate was based on only three studies and should not be interpreted as evidence of true consistency across trials.

**Conclusions:**

Exercise-based prehabilitation was reported as safe and feasible in the included studies, with a possible but non-significant benefit in reducing postoperative pain. Given the low certainty of evidence, further high-quality RCTs are needed to clarify its role in breast cancer surgical care.

**Graphical abstract:**

**Figure:**
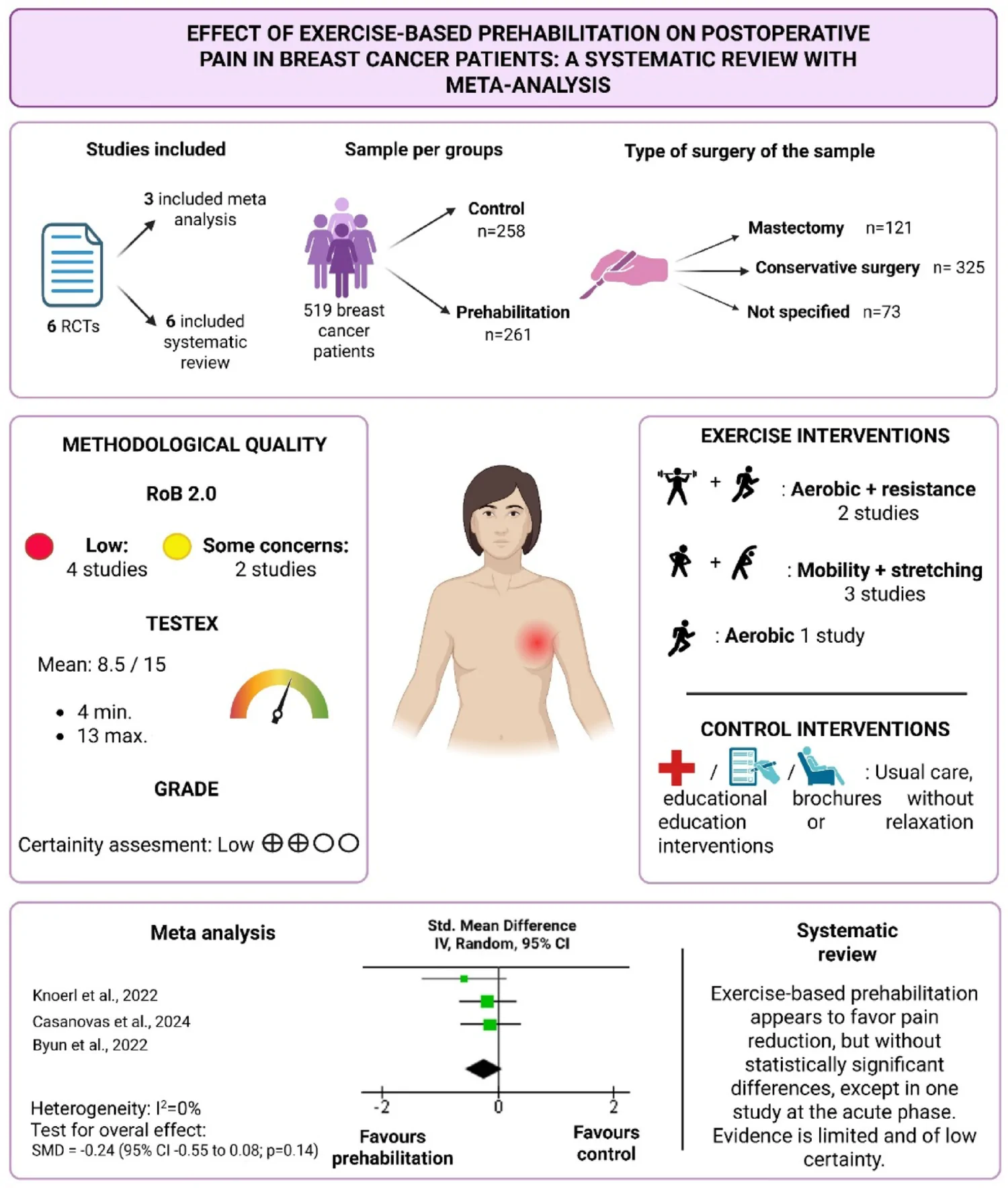

**Supplementary Information:**

The online version contains supplementary material available at https://doi.org/10.1007/s12282-026-01899-4.

## Introduction

Breast cancer remains the most diagnosed cancer among women worldwide and a leading cause of cancer-related morbidity and mortality, with an estimated 2.3 million new cases globally in 2022, accounting for 11.6% of all cancer diagnoses, and approximately 666,000 deaths worldwide [1]. Meanwhile, between 2008 and 2014, breast cancer survival rates in developed countries ranged from approximately 87–90% at 5 years to 78–83% at 10 years, reflecting a notable improvement due to early detection and therapeutic advances [2, 3]. Breast cancer management involves a range of therapeutic modalities aimed to eradicating malignant cells, including radiotherapy, chemotherapy, hormone therapy, and surgical interventions, among others [4]. Such treatments may be delivered as monotherapy or in various multimodal combinations, depending on tumour biology, stage at diagnosis, and patient-specific factors [5, 6]. Surgical interventions remain the primary treatment modalities for breast cancer, typically involving either breast-conserving surgery (lumpectomy) or mastectomy, depending on tumour characteristics and clinical indications [7]. However, a significant proportion of patients may experience acute post-operative pain, a potential complication associated with diminished quality of life [8], increased risk of occupational disability [9], elevated health care utilization and reduced shoulder function [10], which can further impair daily activities and physical rehabilitation [11].

In the last years, regular exercise has become a key part of supportive care in oncology. There is extensive evidence showing that it improves physical function, cardiorespiratory fitness, strength levels, cancer-related fatigue, body composition and psychological well-being in people with different types of cancer at all stages of the disease [12–16]. Specifically, exercise interventions have been shown to have beneficial effects on upper limb mobility, reduce the incidence of lymphedema and improve overall quality of life and functional recovery in breast cancer survivors [17, 18]. These outcomes have led major expert panels, such as those convened by the American College of Sports Medicine [13] or the Consensus Statement from International Multidisciplinary Roundtable [12], to recommend cancer survivors to avoid inactivity and engage in tailored exercise programs during and after treatment. Most interventions have traditionally focused on post-treatment rehabilitation, however, there is increasing interest in introducing structured physical activity or structured exercise before surgical or systemic interventions, a concept known as prehabilitation, with the aim of optimizing patients physical resilience and potentially mitigate postoperative complications [19, 20].

Prehabilitation refers to an intervention that is initiated between cancer diagnosis and the start of acute treatment, which aims to improve patients physical and psychological capacity prior to start of the treatment. It typically includes components such as structured exercise, nutritional optimisation and psychosocial support, all designed to improve initial functional capacity and mitigate postoperative complications [21, 22]. In the field of surgical oncology, especially in cases of major resections, prehabilitation has been associated with better postoperative outcomes, lower complication rates, shorter hospital stays and faster return to baseline function [23, 24]. Despite these promising results, research on its application in breast cancer remains limited. A growing number of pilot studies and qualitative research suggest that exercise-based prehabilitation is feasible and safe, and can support functional recovery, in particular shoulder mobility, improving overall wellbeing [25–28]. However, its specific impact on persistent postoperative pain remains underexplored. This gap is particularly relevant given that persistent post-surgical pain is a frequent complication after breast cancer surgery, affecting approximately 25–60% of patients depending on the definition and assessment method used [29]. To date, two reviews have reported promising findings regarding the impact of exercise-based prehabilitation on postoperative pain in patients undergoing breast surgery [25, 30]. However, the current evidence base consists primarily of narrative syntheses; therefore, quantitative synthesis through meta-analysis is needed to provide more precise estimates of effect and their associated uncertainty.

The main objective of this work is to evaluate the effect of exercise-based prehabilitation on post-operative pain in breast cancer patients. We sought to synthesise the available evidence by means of a systematic review and meta-analysis of randomised clinical trials. The methodological quality of the included studies will also be analysed and the results are intended to guide future clinical recommendations and lines of research. Establishing whether exercise-based prehabilitation reduces postoperative pain could inform preoperative care pathways and potentially reduce the burden of persistent postsurgical pain.

## Methods

This systematic review was carried out following the Preferred Reporting Items for Systematic Reviews and Meta-analysis [31]. The review protocol was registered on PROSPERO (CRD420251016469) on June 16, 2025. The literature search was conducted on June 1, 2025, before protocol registration. Postoperative pain at approximately one month was not explicitly specified as the primary outcome timepoint in the initial PROSPERO record; this timepoint was selected during data extraction, before quantitative synthesis, because it was the most consistently reported postoperative follow-up across eligible studies. The lack of prospective registration before the search process began is acknowledged as a limitation.

### Literature searching strategies

The literature search was conducted in three electronic databases, PubMed, Web of Science and Scopus. The search was conducted on June 1, 2025. Search strategies were structured based on the Population, Intervention, Comparison, Outcome, and Study design framework (PICOS), and included a combination of indexed terms and free-text keywords, linked using Boolean operators to ensure a comprehensive retrieval of relevant studies. The general search phrase as well as the specific search for each database is summarized in Online resource 1. The search was limited to studies published in English, without date restrictions.

### Eligibility criteria

The selection criteria for including or excluding studies were established in accordance with the PICOS framework.

I) **Population**: Women over 18 who have been diagnosed with breast cancer, scheduled for surgery to remove one or two breasts (either a radical or conservative procedure). No restrictions were applied regarding cancer stage or type of breast cancer surgery. When reported, studies including patients with previous breast cancer surgery or contralateral breast surgery were excluded if these participants were not analysed separately; however, this information was not consistently reported across all studies.
II) **Intervention**: Structured preoperative physical exercise programs designed as prehabilitation prior to breast cancer surgery. These programs could include aerobic training, resistance exercises, stretching, mobility or breathing exercises, and may be delivered individually or in combination. Interventions could be provided in person or remotely, and could also include educational components or supervised sessions, as long as physical exercise was a core component of the intervention delivered before surgery.
III) **Comparison**: Control groups receiving usual care, standard preoperative recommendations, educational brochures, or no specific intervention. Comparators were required not to include any structured or supervised physical exercise program prior to surgery. Studies were excluded if the comparator group received an active prehabilitation intervention, or if no control or comparator group was present.
IV) **Outcomes**: Preoperative and postoperative pain. Change scores were calculated as postoperative minus preoperative pain values, with negative values indicating pain reduction.
V) **Study design**: Only RCTs were included. Quasi-experimental studies, cohort studies, and case series were excluded.

Studies meeting all inclusion criteria were included in the quantitative synthesis. Those not fulfilling one or more criteria were integrated into the qualitative synthesis to provide additional context.

### Studies selection and data extraction

Study selection was performed in two stages: an initial screening based on titles and abstracts, followed by a full-text review to assess eligibility according to the predefined inclusion criteria. Data from eligible studies were extracted and organized in an Excel spreadsheet. The variables collected included publication year, authors, sample size, type of surgery, study design, type and duration of the intervention, characteristics of the control group and the measurement instrument used for pain. For studies reporting multiple pain measures (e.g., both VAS and EORTC QLQ-C30), the measure designated as the primary outcome by the study authors was extracted when available; otherwise, the most clinically relevant measure was selected. During the data extraction phase, and prior to any quantitative synthesis, we predefined the extraction of postoperative pain at approximately one month as the target outcome. Of the six included studies, four reported pain-related outcomes at approximately one month post-surgery, making it the most commonly reported postoperative timepoint. Preoperative pain values were extracted to calculate pre-to-postoperative change scores. This process was carried out independently by two authors (Asier del Arco and Jose Pinto-Fraga). In case of discrepancies, a third author (Arkaitz Castañeda-Babarro) was consulted.

### Risk of bias and evidence certainty assessments

The methodological quality and potential risk of bias were assessed using the standardized Cochrane Collaboration risk-of-bias tool (RoB 2) [32]. Each study was evaluated across five domains: bias arising from the randomization process, bias due to deviations from intended interventions, bias due to missing outcome data, bias in the measurement of the outcome, and bias in the selection of the reported result. For each domain, studies were classified as having “low risk,” “some concerns,” or “high risk” of bias, and an overall risk-of-bias judgment was subsequently derived in accordance with the RoB 2 guidance [32].

On the other hand, the tool proposed by Grading of Recommendations Assessment, Development and Evaluation (GRADE) was used to assess the certainty of the evidence [33]. The quality of the prehabilitation intervention results was assessed using five different criteria: study design, risk of bias, imprecision, inconsistency, and indirectness. Based on these standards, the evidence was considered to be of high, moderate, low, or very low certainty. The GRADE assessment was performed for the primary outcome only; secondary outcomes and studies not included in the meta-analysis were not formally assessed for certainty of evidence.

Finally, in accordance with PRISMA guidelines [31], the risk of bias analysis was supplemented by an evaluation of methodological quality. This methodological assessment was carried out using the The Tool for the assessment of study quality and reporting in exercise (TESTEX) [34], designed specifically to assess the methodological rigor and reporting standards of physical exercise interventions. The TESTEX scale includes 12 criteria and provides a maximum score of 15 points, divided into two core areas: 5 points for study quality and 10 points for reporting quality. This assessment tool covers the following aspects: (1) clearly defined and met eligibility criteria; (2) detailed and specified randomization procedures; (3) allocation concealment for participants; (4) groups statistically similar at baseline; (5) evaluator blinding; (6 A) over 85% of participants completed the study; (6B) adverse events reported per group; (6 C) session attendance reported for study completers; (7) use of intention-to-treat analysis; (8 A) statistical comparisons between groups for the primary outcome; (8B) between-group comparisons for secondary outcomes; (9) reporting of outcome variability; (10) physical activity levels of the control group disclosed; (11) exercise intensity modified as needed during the intervention; and (12) ability to calculate total exercise volume and energy expenditure. The TESTEX scale was selected because it specifically addresses methodological features relevant to exercise interventions, such as exercise intensity progression and session attendance reporting, which are not captured by generic quality assessment tools. These 3 processes concerning the assessment of risk of bias, the quality of exercise interventions or the evaluation of certainty were carried out by the same authors as the screening process.

Two reviewers (Asier del Arco and Jose Pinto-Fraga) independently carried out this process, and any disagreements were resolved by consultation with a third reviewer (Arkaitz Castañeda-Babarro).

### Meta‑analysis

Data was meta-analized using RevMan 5.4. The mean and standard deviation of the outcome indicators were extracted. The standardized mean difference (SMD), calculated using Hedges’g to account for small sample bias, with 95% confidence intervals (CI), was used to estimate the pooled effect size. For the meta-analysis, effect sizes were calculated using pre-to-postoperative change scores. Heterogeneity was assessed using the I² statistic and P value from the Q test: low heterogeneity (I² ≤ 50% and *P* ≥ 0.1), moderate heterogeneity (50% < I² < 75% and *P*< 0.1), and high heterogeneity (I²> 75% and *P*< 0.1).

A random-effects model was employed to account for the expected variability across studies in terms of intervention components, delivery formats, and outcome measurement. However, given that only three studies were included in the meta-analysis, the between-study variance (tau²) was estimated with substantial imprecision and should therefore be interpreted cautiously. This approach aligns with methodological guidance for meta-analyses in exercise science, where heterogeneity is typically assumed due to the inherent diversity in study designs [35]. Importantly, the use of a random-effects model is considered appropriate even when statistical heterogeneity appears low, as it better reflects the assumption that included studies estimate different, yet related, true effects. When studies reported multiple postoperative timepoints, the measurement closest to 30 days post-surgery was extracted. Studies were considered eligible for quantitative synthesis if they reported postoperative pain outcomes at approximately one month and provided sufficient data (mean and standard deviation) to calculate standardized effect sizes. No data transformations or imputations were performed and no subgroup or meta-regression analyses were performed due to the small number of included studies in the quantitative synthesis. All included studies reported pain outcomes as means and standard deviations; no studies were excluded for reporting medians and interquartile ranges. No continuity corrections were applied because all outcomes were continuous variables. Analyses were conducted using RevMan (v. 5.4), in accordance with Cochrane methodological standards. Reporting bias and certainty of evidence were assessed using a funnel plot and the GRADE approach, respectively.

Authors of studies not included in the quantitative synthesis were not contacted for additional data because exclusion was due to predefined methodological incompatibility criteria: lack of a comparable one-month postoperative pain assessment (*n* = 1), pain outcomes not reported as stand-alone data suitable for effect-size calculation (*n* = 1), or absence of a preoperative pain assessment required to calculate pre-to-postoperative change scores (*n* = 1). An exploratory post-hoc sensitivity analysis excluding Knoerl et al. was conducted using the same random-effects model as the primary analysis.

## Results

**Fig. 1:**
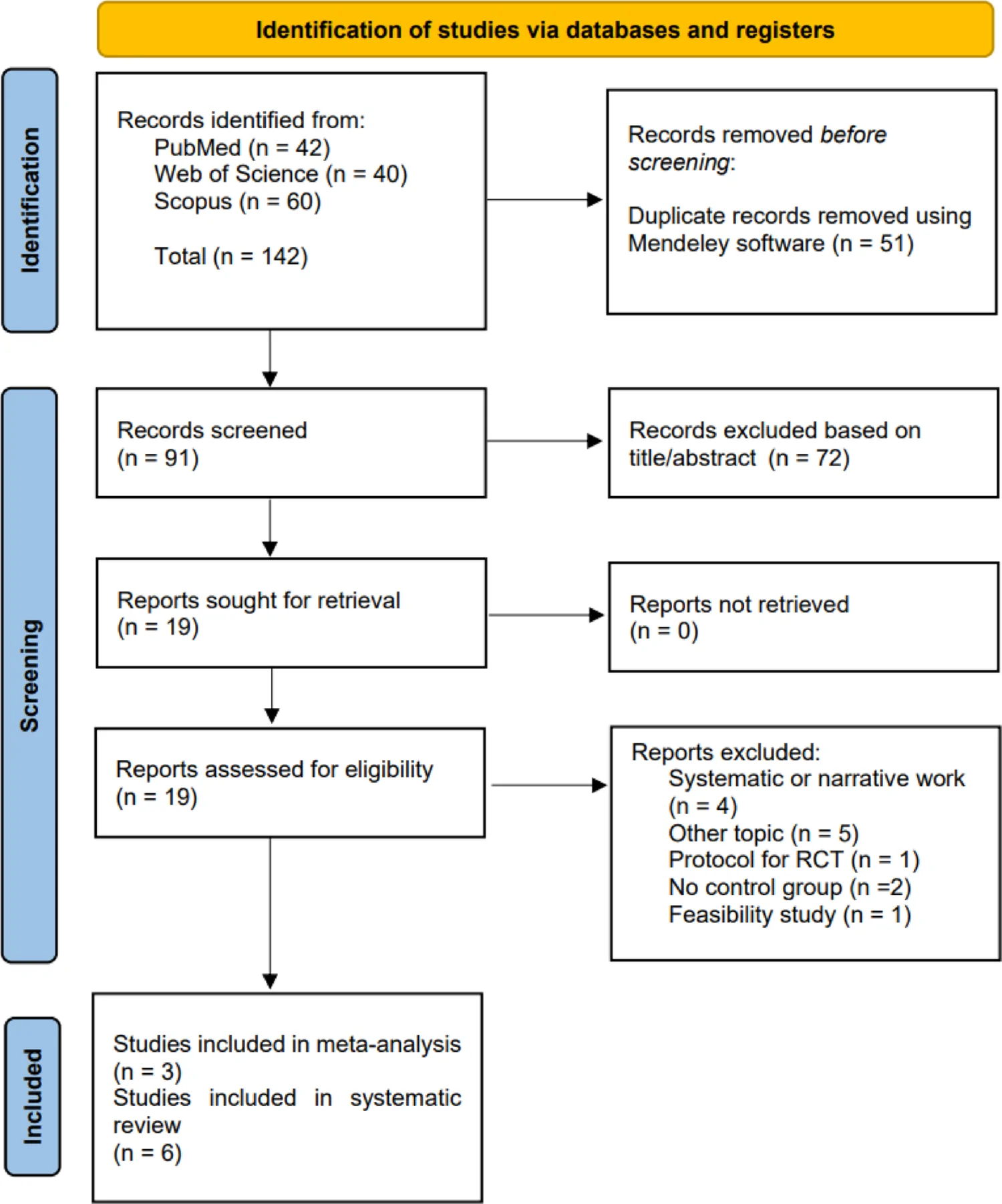
Flowchart of studies selection criteria

Literature searching and studies selection process was carried out in accordance to PRISMA guidelines as shown in Fig. 1. The search phrase yielded a total of 142 results, of which 51 were duplicates (identified using Mendeley software). The titles and abstracts of the remaining 91 papers were evaluated, of which 72 were eliminated. After this screening, a complete read of the remaining 19 articles was carried out, excluding 13 for different reasons, as can be seen in the Fig. 1 flow chart.

### Characteristics of included studies

The main results of the studies included in the review, including number of participants, type of surgery, instrument used, and main outcomes, are summarised in Table 1. Of the included RCTs, one was conducted in the United States [36], one in Spain [37], one in Norway [38], one in India [39], one in Pakistan [40] and one in South Korea [41].

**Table 1:** General characteristics of studies included in the systematic review and meta-analysis

Authors and year	Sample	Surgery type	Evaluation tool	Results	Included in quantitative synthesis	Reason for excluding from meta-analysis
Byun et al., 2022	61 (E: 31 C: 30)	BCS: 46 Mastectomy: 15	VAS	Between group (PH vs. CG): Not significant. Within group (Preop vs. Postop) E: Significant. C: Significant	Yes	Not applicable
Casanovas-Álvarez et al., 2024	64 (E: 32 C: 32)	BCS: 25 Mastectomy: 27 N.R.: 12	EORTC-QLQ-C30	Between group (PH vs. CG): Not significant. Within group (Preop vs. Postop): E: Significant. C: Significant.	Yes	Not applicable
Knoerl et al., 2022	47 (E: 26 C:21 )	BCS: 27 Mastectomy: 19 N.R.: 1	EORTC-QLQ-C30	Between group (PH vs. CG): Not significant. Within group (Preop vs. Postop) E: Not significant. C: Not significant.	Yes	Not applicable
Lokapavani et al., 2014	30 (E: 15 C:15)	N.R.	SPADI	The pain and disability scores increased significantly in the control group compared to the prehabilitation group.	No	Pain reported together with disability and not available as a stand-alone outcome.
Fatima et al., 2022	30 (E: 15; C:15)	N.R.	NPRS	The prehabilitation group had less pain in the early postoperative phase.	No	No comparable postoperative pain assessment at approximately one month.
Heinman et al., 2022	287 (E: 139; C: 148)	BCS: 227 Mastectomy: 60	RAND-36	There was no difference between the groups for postoperative pain.	No	No preoperative pain assessment available to calculate pre-to-postoperative change scores.

N.R. = not reported; VAS = Visual Analogue Scale; NPRS = Numeric Pain Rating Scale; PH = prehabilitation group; CG = control group; E = experimental group; C = control group; BCS = breast-conserving surgery

For studies included in the quantitative synthesis, ‘Within group (Preop vs. Postop)’ refers to the comparison between preoperative pain and pain at the postoperative follow-up timepoint closest to one month. ‘Between group (PH vs. CG)’ refers to the comparison between the prehabilitation and control groups at the same postoperative timepoint. Within-group (Preop vs. Postop) and between-group (PH vs. CG) comparisons are reported independently and should be interpreted separately. For example, “E: Significant; C: Significant” indicates that both the experimental and control groups showed a significant within-group change from preoperative to 1-month postoperative assessment.

### Characteristics of participants

The total sample of the included studies consisted of 519 women diagnosed with breast cancer, of whom 55.3% belonged to a single study [38]. Of these, 258 (49,7%) belonged to the prehabilitation group, while 261 (50,3%) belonged to the control group. Four of the studies reported the types of surgery performed [36–38, 41], with two of them reporting some patients in whom this was not specified [36, 37]. However, two of the studies did not report this information [39, 40]. With regard to surgery, 325 underwent conservative surgery, 121 received mastectomy, and for 73 patients the type of surgery was not specified.

### Characteristics of interventions

Regarding the instruments used to assess pain outcomes, the included studies employed a variety of validated tools, such as VAS, EORTC QLQ-C30 (pain subscale), SPADI, NPRS or RAND36 [36–40]. The duration of prehabilitation interventions varied across studies, ranging from a single preoperative session to 6–9 weeks, with most programs lasting between 1 and 4 weeks [36–40]. The exercise-based prehabilitation interventions varied across studies in terms of content and structure, but all incorporated elements aimed at improving physical function before surgery and their main characteristics can be appreciated in Table 2. Control group interventions also varied across studies but generally consisted of standard care, minimal education, or routine recommendations. Two studies provided only printed or verbal educational materials [39, 41]. Other two researches offered usual oncological care [37, 38], while another study included a self-directed program as the comparator [36].

### Methodological quality and risk of bias results

**Fig. 2:**
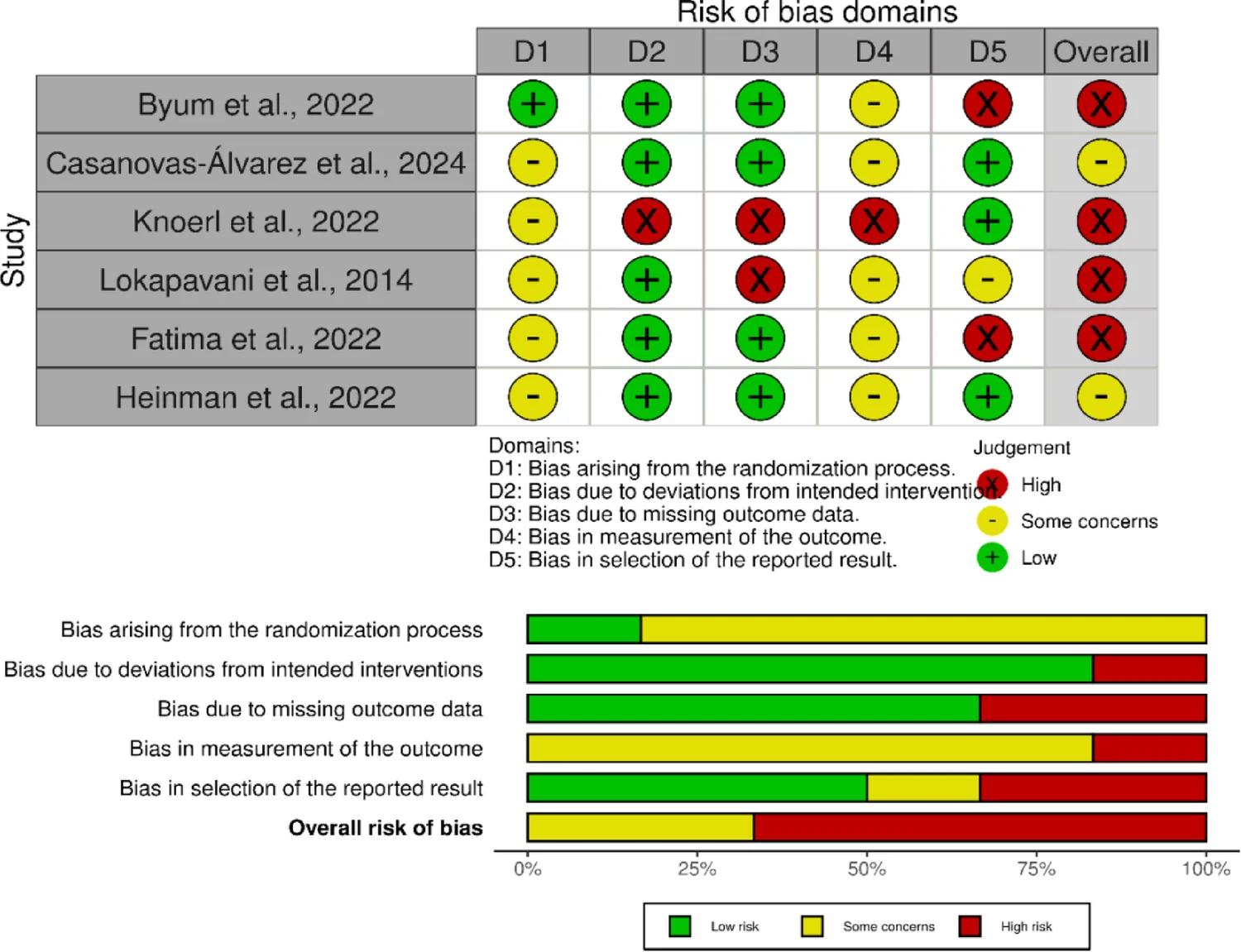
Risk of bias of the studies included in the review

The risk of bias assessment using the Cochrane RoB 2 tool is presented in Fig. 2. Detailed domain-level RoB 2 judgments, including the specific judgments for each study across all five domains, are presented in Online resource 2. A summary of the main methodological concerns for each study is also provided. Most concerns regarding bias were identified in the domains of the randomization process particularly due to the lack of clarity in allocation concealment and the absence of assessor blinding [36, 38–41] as well as potential influence of knowledge of the intervention on outcome evaluation [36–41]. However, the risk of bias was low in domains related bias due to deviations from intended intervention and bias due to missing outcome data, except for Knoerl [36] and Lokapavani [39].

**Table 2:** Characteristics of exercise-based prehabilitation and control interventions of included studies

Authors and year	Duration	Control intervention	Prehabilitation intervention
Byum et al., 2022	Single session before surgery	Self-reading of educational brochures on lymphedema prevention and shoulder pain relief exercises.	Same brochures + 1:1 education about the exercise program. Verbal and physical comprehension check. Additional 30 min re-education on unclear parts.
Casanovas-Alvarez et al., 2024	6–9 weeks during neoadjuvant therapy	Usual care: oncology visits and on-demand counselling.	Supervised Nordic Walking + resistance training (2x/week, 75 min/session). Includes health education and relaxation.
Knoerl et al., 2022	4 weeks (mean: 28.9 days).	Mind–body self-directed program (relaxation, breathing, guided imagery).	Supervised aerobic + resistance training (2x/week, 60–90 min/session). At-home aerobic activity encouraged.
Lokapavani et al., 2014	1–2 weeks.	Pre-op educational brochure.	Supervised Exercise (3x/week) including mobility, breathing, relaxation, self-stretching and walking program.
Fatima et al., 2022	1–2 weeks.	No preoperative intervention.	2–5 sessions per week of static shoulder stretches and mobility.
Heinman et al., 2022	2 weeks.	Usual care.	Aerobic activity 30/min day 7x/week.

As shown in Fig. 2, concerns related to measurement of the outcome were present in all six studies, while concerns related to the randomization process were present in five of the six studies. Four studies were judged to be at low risk of bias for missing outcome data, whereas three were judged to be at low risk for selection of the reported result.

The methodological quality of exercise intervention from the included trials, evaluated using the TESTEX checklist, is summarized in Table 3. The TESTEX scores ranged from 4 to 13 out of 15, with a median score of 6.5. The low scores reflect common methodological limitations in exercise intervention studies, including inadequate blinding, lack of adverse event reporting, and insufficient description of exercise intensity progression. Of the six studies assessed, only one achieved a score greater than 10/15 [37], while the remaining five scored between 4 and 10 points [36, 38–41].

**Table 3:** Methodological quality assessment of exercise-based interventions using the TESTEX scale

Author and year	1	2	3	4	5	6 A	6B	6 C	7	8 A	8B	9	10	11	12	Total
Byum et al., 2022	1	1	1	1	1	1	0	0	0	1	1	1	0	0	0	9
Casanovas-Alvarez et al., 2024	1	1	1	1	1	1	1	1	1	1	1	1	1	0	0	13
Knoerl et al., 2022	1	1	1	1	0	0	0	0	0	1	1	1	1	0	1	9
Lokapavani et al., 2014	1	0	1	0	0	0	0	0	0	1	1	0	0	0	0	4
Fatima et al., 2022	1	1	1	N.A.	0	1	0	0	0	1	1	1	0	0	0	6
Heinman et al., 2022	1	0	0	1	0	0	1	1	1	1	1	1	1	0	1	10

1 = Eligibility criteria clear and met; 2 = Randomization methods described and defined; 3 = Concealment of participant assignment; 4 = Groups with no statistical difference in pretest; 5 = Blinding of evaluator; 6 A = More than 85% of participants completed the study; 6B = Adverse events reported for each group; 6 C = Attendance at completed sessions reported for participants who completed the study; 7 = Intention-to-treat analysis; 8 A = Between-group statistical analysis is reported for the primary dependent variable; 8B = Between-group statistical analysis is reported for the secondary variable(s); 9 = Outcome variability results are reported; 10 = Physical activity level of the control group is reported; 11 = Exercise intensity was adjusted during the intervention; 12 = Volume and energy expenditure can be calculated; N.A. = Not applicable

Overall, studies scoring below 10 points presented several methodological limitations, particularly related to evaluator blinding, reporting of adverse events, and progression of exercise intensity during the intervention [36, 38–41]. In particular, the study by Lokapavani et al. [39], which scored 4 out of 15, should be interpreted as having very poor methodological quality, and conclusions drawn from this study should therefore be considered with caution. The only study scoring above 10 [37] incorporated supervised sessions, reported attendance, and described exercise progression, methodological features that may enhance the rigor and reproducibility of exercise-based interventions.

### Studies not included in meta-analysis

Among the studies not included in the quantitative synthesis, Lokapavani et al. [39] reported greater improvements in the prehabilitation group compared with the control group in combined pain and disability outcomes between the postoperative assessment and one-month follow-up. These findings suggested better functional recovery and fewer symptoms among participants receiving preoperative physiotherapy education and exercise.Fatima et al. [40] found significantly lower postoperative pain in the prehabilitation group compared with the control group during the early postoperative period, specifically at one and three days after surgery. However, this effect was reported only in the acute postoperative phase. Heiman et al. [38] reported no significant differences between groups in postoperative pain outcomes. Overall, the findings from studies not included in the meta-analysis were mixed, with two studies suggesting short-term or functional benefits associated with prehabilitation [39, 40], whereas one study reported no between-group difference [38]. However, these results should be interpreted with caution due to the methodological limitations of these studies, whose TESTEX scores ranged from 4 to 10.

### Studies included in meta-analysis

**Fig. 3:**
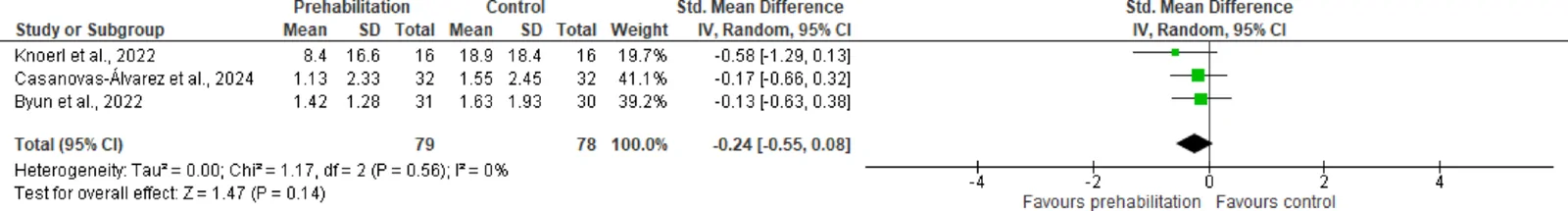
Forest plot of studies included in the meta-analysis

*Notes.* The I² estimate was 0% (95% CI: 0% to 79%) and was based on only three studies. The wide confidence interval indicates that the true heterogeneity could range from none to substantial; therefore, the absence of statistical heterogeneity should not be interpreted as evidence of true consistency across trials.

The studies included in the quantitative synthesis were three randomized controlled trials with heterogeneous prehabilitation interventions in terms of duration, modality, and supervision, and presented mainly some concerns or high risk of bias according to the RoB 2 assessment [36, 37, 41]. The other three studies were excluded for different reasons, such as reporting data together with other scales and not in stand-alone form [39], not having preoperative measurement [38] or not reporting a comparable postoperative timepoint [40]. Following the meta-analysis results (Fig. 3), prehabilitation interventions yielded a small, non-significant standardized mean difference on pain reduction compared to controls (pooled SMD = -0.24; 95% CI (-0.55, 0.08); *p* = 0.14). Although statistical heterogeneity was low (I² = 0%; 95% CI: 0% to 79%; *p* = 0.56), the confidence interval was extremely wide, indicating that the true heterogeneity could range from none to substantial. With only three studies, neither the I² statistic nor the heterogeneity test can reliably distinguish true homogeneity from insufficient statistical power to detect heterogeneity. Therefore, this result should not be interpreted as evidence of consistency across trials, particularly given the clinical differences in intervention duration, exercise modality, supervision, and pain assessment tools.

An exploratory post-hoc sensitivity analysis was performed to assess the impact of Knoerl et al. [36], which was selected because it had the largest standard deviation (SD = 16.6) relative to its mean (M = 8.4), potentially indicating skewness or influential values, although no formal outlier analysis was conducted. This sensitivity analysis used the same random-effects model as the primary analysis. After excluding this study, the pooled effect size remained non-significant (SMD = -0.15; 95% CI: -0.50 to 0.20; *p* = 0.40; I² = 0%). These findings suggest that the exploratory exclusion of this study did not materially affect the overall results (Online resource 3). A funnel plot was generated and is presented as Online resource 4. Current methodological guidance recommends a minimum of 10 studies for meaningful funnel plot interpretation [42]. No meaningful interpretation of publication bias is possible with only three studies; this figure is provided for completeness only and should not be used to infer absence of publication bias.

### Certainty of evidence

Using the GRADE framework, the certainty of evidence for the primary outcome was rated as low (Online resource 5). Evidence was downgraded due to serious risk of bias and imprecision. Risk of bias was considered serious primarily because concerns related to measurement of the outcome were present in all six included studies, while concerns related to the randomization process were present in five of the six studies. Regarding imprecision, the pooled estimate crossed the null value (SMD = − 0.24; 95% CI: −0.55 to 0.08), encompassing both no effect and a possible small benefit. Moreover, an optimal information size based on an SMD of 0.30, α = 0.05, and β = 0.20 would require approximately 350–400 participants, whereas the meta-analysis included only 157 participants. Inconsistency was not formally downgraded because statistical heterogeneity was low (I² = 0%); however, this estimate is unreliable because only three studies were included. Evidence was not downgraded for indirectness because the included populations, interventions, comparators, and pain outcomes were considered directly applicable to the review question.

## Discussion

The primary objective of this systematic review and meta-analysis was to assess the impact of physical exercise prehabilitation on postoperative pain. Exercise-based prehabilitation yielded a small but non-significant effect on postoperative pain at one month following breast cancer surgery. The pooled effect was small in magnitude (SMD = − 0.24). However, its clinical relevance remains uncertain because no outcome-specific minimal clinically important difference was prespecified and different pain assessment instruments were used across studies. Although the standardized mean difference did not reach statistical significance, the observed non-significant trend may be due to chance, particularly given the small number of included studies and the low certainty of evidence. These results must be interpreted cautiously, particularly considering the methodological limitations of the included studies.

It is important to note that postoperative pain is a multifaceted phenomenon involving both nociceptive and neuropathic components, with potential transition into chronic pain if not adequately addressed [43]. In breast cancer patients, pain may be driven by tissue damage, inflammation, nerve injury, and psychological distress [44]. Exercise-based interventions may modulate pain through several pathways, including reduction of systemic inflammation [45]. Beyond physiological mechanisms, exercise-based prehabilitation may also influence postoperative pain through psychosocial pathways, including reductions in pain catastrophizing and improvements in self-efficacy, which are known to modulate pain perception [45]. However, the exact biological mechanisms by which prehabilitation may affect pain outcomes, particularly whether they differ in acute vs. chronic contexts, remain insufficiently understood and warrant further investigation.

The findings are consistent with previous qualitative reports suggesting better outcomes with exercise-based prehabilitation, although without statistically significant differences [25, 30]. However, quantitative evidence on its direct impact on acute or persistent postoperative pain remains scarce. This lack of conclusive evidence may be partly explained by variability across the included trials in intervention protocols, study designs, delivery format, supervision, exercise components, and outcome measurement tools [36–41]. Interventions ranged from brief or single-session approaches to longer supervised programs, and pain was assessed using different instruments, including the VAS [41], NPRS [40], EORTC QLQ-C30 pain subscale [36, 37], SPADI [39], and RAND-36 [38]. Therefore, the pooled effect estimate should be interpreted as an average effect across heterogeneous exercise-based prehabilitation approaches rather than as evidence of modality-specific efficacy.

The methodological quality of the studies was generally low to moderate. Only one study exceeded 10 points on the TESTEX scale, and several studies presented some concerns or high risk of bias according to RoB 2, particularly in relation to measurement of the outcome and the randomization process. This aligns with previous research [46], which also noted the overall low methodological quality of prehabilitation trials in oncology. The implementation of core outcome sets, including standardized pain scales and consistent follow-up time points, would improve comparability across future trials.

Despite the current lack of statistically significant evidence, the feasibility and safety of exercise-based prehabilitation support its further investigation as a potential adjunct in the surgical management of breast cancer [21, 27]. Importantly, the direction of effect was consistent across the studies included in the meta-analysis, with all favouring prehabilitation, although none demonstrated statistically significant benefits [46, 47]. One study [40] reported a statistically significant reduction in pain; however, this effect was observed in the acute postoperative period (1–3 days), a time point that was not assessed in any of the other five included studies [36–39, 41]. Conversely, Hayashi et al. [48] reported improvements in pain outcomes over longer follow-up periods across several surgical populations, suggesting that intervention timing and duration may influence outcomes. However, these findings were not specific to patients undergoing breast cancer surgery and should not be directly extrapolated to this population. In addition to its potential impact on pain, exercise-based prehabilitation may offer other meaningful benefits, including shorter hospital stays and enhanced physical readiness for treatment [49]. Studies have also reported gains in shoulder range of motion [50] and even immune system modulation in response to preoperative exercise [51]. These physiological responses are consistent with emerging evidence on how exercise may influence cancer hallmarks [52].

This review is limited by the small number of eligible randomized trials and their generally modest methodological quality. Only three studies, including 157 participants, were included in the meta-analysis, which restricted statistical power, limited the reliability of heterogeneity estimates, and prevented meaningful subgroup analyses or direct comparisons between exercise modalities. With this sample size, the meta-analysis was only adequately powered to detect approximately moderate effects, around an SMD of 0.45, but not small effects in the range of 0.2–0.3. Although clinical heterogeneity was present due to differences in intervention type, duration, delivery format, follow-up timing, and pain assessment tools, statistical heterogeneity was not detected, likely reflecting the limited power of the analysis rather than true homogeneity. Reporting of adherence and safety was often insufficient, and potentially relevant participant-level factors, such as age, could not be examined as modifiers of the observed effects due to limitations in reporting and data availability across the included studies. Another limitation emerged during the review process, as postoperative pain at approximately one month was selected as the primary outcome timepoint during data extraction, before quantitative synthesis. The selection of this timepoint was based on data availability rather than a prespecified clinical rationale, and its clinical relevance for predicting longer-term pain outcomes remains unknown. Therefore, this timepoint should be interpreted as a data-driven methodological decision rather than as a clinically prespecified endpoint. In addition, although both the database search date and PROSPERO registration date were transparently reported, the search was conducted before PROSPERO registration, and therefore the review was not prospectively registered before the search process began. The certainty of evidence was assessed only for the primary outcome included in the meta-analysis, and not for secondary outcomes or for studies excluded from the quantitative synthesis. Furthermore, potential conflicts of interest and funding sources of the included studies were not systematically assessed. The limited number of studies also prevented the assessment of potential publication bias through funnel plot asymmetry tests, which require a minimum of 10 studies for meaningful interpretation [42]. Finally, the search was limited to PubMed, Scopus, and Web of Science, and did not include grey literature sources or trial registries, which may have increased the risk of publication bias.

In conclusion, exercise-based prehabilitation was reported as safe and feasible in the included studies, but showed only a small, non-significant reduction in postoperative pain that does not rule out the possibility of no effect. The available evidence is insufficient to support the routine implementation of exercise-based prehabilitation specifically for pain reduction in breast cancer surgery. However, the safety and feasibility reported in the included studies suggest that further investigation is warranted.

To strengthen the current evidence base, future research should first prioritize adequately powered randomized trials with blinded outcome assessment to detect clinically meaningful differences in postoperative pain. Moreover, exercise-based prehabilitation protocols should be more clearly defined in terms of type, frequency, intensity, duration, and level of supervision. The use of standardized outcome measures, including pain assessed at consistent follow-up points such as 1 and 3 months post-surgery, would enhance comparability across studies. Finally, systematic reporting of adherence and adverse events is essential to reduce bias, improve transparency, and facilitate implementation in clinical practice.

## Supplementary Information

Below is the link to the electronic supplementary material.

Supplementary Material 1

Supplementary Material 2

Supplementary Material 3

Supplementary Material 4

Supplementary Material 5

## Data Availability

The data used in the present study are available upon request from the authors of the articles included in the review.

## Data availability

Data extracted from included studies are available from the corresponding author upon reasonable request. This review did not generate new data.
