# Supplementary material for: Effect of exercise based prehabilitation on postoperative pain in breast cancer patients: a systematic review with meta-analysis
Source: Breast Cancer. 2026 Jul 30;33(5):964–77. doi: 10.1007/s12282-026-01899-4 (PMC13522077; doi:10.1007/s12282-026-01899-4)
Supplement: Supplementary file 1 [file 12282_2026_1899_MOESM1_ESM.docx]

**Online resource 1.** Database specific searches.

**Pubmed**

(("Muscle Stretching Exercises"[Mesh]) OR ("Range of Motion, Articular"[Mesh]) OR (Exercise[Mesh]) OR ("Physical Therapy Modalities"[Mesh]) OR (exercise ) OR ("physical activity")) AND (("Preoperative Exercise"[Mesh]) OR (prehabilitation) OR (preoperative)) AND (("Breast Neoplasms"[Mesh]) OR ("breast cancer")) AND (("Pain, Postoperative"[Mesh]) OR ("Shoulder Pain"[Mesh]) OR (pain) OR ("shoulder function")).

**Web Of Science**

(("Muscle Stretching Exercises") OR ("Range of Motion") OR (Exercise) OR ("Physical Therapy Modalities") OR (exercise ) OR ("physical activity" )) AND (("Preoperative Exercise") OR (prehabilitation ) OR (preoperative)) AND (("Breast Neoplasms") OR ("breast cancer")) AND (("Pain, Postoperative") OR ("Shoulder Pain") OR (pain) OR ("shoulder function")).

**Scopus**

(("Muscle Stretching Exercises") OR ("Range of Motion") OR (Exercise) OR ("Physical Therapy Modalities") OR (exercise ) OR ("physical activity")) AND (("Preoperative Exercise") OR (prehabilitation ) OR (preoperative)) AND (("Breast Neoplasms") OR ("breast cancer")) AND (("Pain, Postoperative") OR ("Shoulder Pain") OR (pain) OR ("shoulder function")).

Database search: June 1, 2025.
