# Supplementary material for: Effect of exercise based prehabilitation on postoperative pain in breast cancer patients: a systematic review with meta-analysis
Source: Breast Cancer. 2026 Jul 30;33(5):964–77. doi: 10.1007/s12282-026-01899-4 (PMC13522077; doi:10.1007/s12282-026-01899-4)
Supplement: Supplementary file 2 [file 12282_2026_1899_MOESM2_ESM.docx]

| **Study** | **D1** | **D2** | **D3** | **D4** | **D5** | **Overall risk of bias** | **Main concerns** |
| --- | --- | --- | --- | --- | --- | --- | --- |
| Byun et al., 2022 | Low | Low | Low | Some concerns | High | High | Concerns related to outcome measurement and selection of the reported result. |
| Casanovas-Álvarez et al., 2024 | Some concerns | Low | Low | Some concerns | Low | Some concerns | Some concerns related to the randomization process and outcome measurement. |
| Knoerl et al., 2022 | Some concerns | High | High | High | Low | High | Concerns related to deviations from intended interventions, missing outcome data, and outcome measurement. |
| Lokapavani et al., 2014 | Some concerns | Low | High | Some concerns | Some concerns | High | Concerns related to missing outcome data, outcome measurement, and selection of the reported result. |
| Fatima et al., 2022 | Some concerns | Low | Low | Some concerns | High | High | Concerns related to outcome measurement and selection of the reported result. |
| Heiman et al., 2022 | Some concerns | Low | Low | Some concerns | Low | Some concerns | Some concerns related to the randomization process and outcome measurement. |

**Online resource 2.** Domain-level RoB 2 judgments and main methodological concerns.for each included study

***Note.*** D1: bias arising from the randomization process; D2: bias due to deviations from intended interventions; D3: bias due to missing outcome data; D4: bias in measurement of the outcome; D5: bias in selection of the reported result; RoB 2: revised Cochrane risk-of-bias tool for randomized trials.
