# Supplementary material for: Effect of exercise based prehabilitation on postoperative pain in breast cancer patients: a systematic review with meta-analysis
Source: Breast Cancer. 2026 Jul 30;33(5):964–77. doi: 10.1007/s12282-026-01899-4 (PMC13522077; doi:10.1007/s12282-026-01899-4)
Supplement: Supplementary file 5 [file 12282_2026_1899_MOESM5_ESM.docx]

**Online resource 5.** GRADE certainty assessment.

| Studies (desing) | Risk of bias | Incosistency | Indirectness | Imprecision | Publication bias | Overall sample size | Effect SMD (95% CI) | Certainty |
| --- | --- | --- | --- | --- | --- | --- | --- | --- |
| Three (RCTs) | Serious ↓ | Not serious | Not serious | Serious ↓ | Not serious | 157 | -0.24  (-0.55, 0.08) | Low ⊕⊕_○○_ |
