# Supplementary material for: Effect of exercise based prehabilitation on postoperative pain in breast cancer patients: a systematic review with meta-analysis
Source: Breast Cancer. 2026 Jul 30;33(5):964–77. doi: 10.1007/s12282-026-01899-4 (PMC13522077; doi:10.1007/s12282-026-01899-4)
Supplement: Supplementary file 6 [file 12282_2026_1899_MOESM3_ESM.docx]

**Online resource 3.** Forest plot of the sensitivity analysis excluding Knoerl et al.
